# A Reliable High-Throughput Screening Model for Antidepressant

**DOI:** 10.3390/ijms22179505

**Published:** 2021-09-01

**Authors:** Rui Zhang, Caili Qiao, Qiuyan Liu, Jingwen He, Yifan Lai, Jing Shang, Hui Zhong

**Affiliations:** 1 State Key Laboratory of Natural Medicines, China Pharmaceutical University, Nanjing 210009, China; zhang_rui1995@outlook.com (R.Z.); Caili_Qiao@outlook.com (C.Q.); 3121020096@stu.cpu.edu.cn (Q.L.); 3219020484@stu.cpu.edu.cn (J.H.); Yifan_Lai@outlook.com (Y.L.); 2 School of Traditional Chinese Pharmacy, China Pharmaceutical University, Nanjing 211198, China

**Keywords:** depression, reserpine-induced model, metabolomics

## Abstract

Depression is the most frequent affective disorder and is the leading cause of disability worldwide. In order to screen antidepressants and explore molecular mechanisms, a variety of animal models were used in experiments, but there is no reliable high-throughput screening method. Zebrafish is a common model organism for mental illness such as depression. In our research, we established chronic unpredictable mild stress (CUMS) models in C57BL/6 mice and zebrafish; the similarities in behavior and pathology suggest that zebrafish can replace rodents as high-throughput screening organisms. Stress mice (ip., 1 mg/kg/d, 3 days) and zebrafish (10 mg/L, 20 min) were treated with reserpine. As a result, reserpine caused depression-like behavior in mice, which was consistent with the results of the CUMS mice model. Additionally, reserpine reduced the locomotor ability and exploratory behavior of zebrafish, which was consistent with the results of the CUMS zebrafish model. Further analysis of the metabolic differences showed that the reserpine-induced zebrafish depression model was similar to the reserpine mice model and the CUMS mice model in the tyrosine metabolism pathway. The above results showed that the reserpine-induced depression zebrafish model was similar to the CUMS model from phenotype to internal metabolic changes and can replace the CUMS model for antidepressants screening. Moreover, the results from this model were obtained in a short time, which can shorten the cycle of drug screening and achieve high-throughput screening. Therefore, we believe it is a reliable high-throughput screening model.

## 1. Introduction

Depression is accompanied by long-lasting cognitive impairment and behavior changes, which can lead to serious socioeconomic and health burdens. The pathogenesis of depression is complex and animal models are essential tools in depression research. Depressed animals exhibit behavioral changes such as depression and cognitive dysfunction, which can mimic the symptoms of depression in humans [1,2,3,4]. Using animal models can not only circumvent the ethical problems of human depression research, but also obtain sufficient sample size for research.

Rodents are the main experimental animals for depression research due to their advantages of stable modeling, easy access and low price [5,6,7]. Commonly used rodent depression models include stress models and drug models. The neurological, endocrine and behavioral changes in the olfactory bulb resection model can mimic human depression by surgical removal or destruction of the olfactory bulb, and the mechanism may be related to the decrease in 5-HT and epinephrine, but it is not suitable for the detection of rapid-acting antidepressants [8]. The social defeat stress model simulates the depressive symptoms of human anhedonia, but only 50% of the mice show depressive symptoms, so a large sample is needed [9]. The learned helplessness model reduced the spontaneous activity of mice, and metabolomics analysis found that it is related to amino acid metabolism, lipid metabolism and sugar metabolism. However, this model can only detect the antidepressant effect of the compound, but cannot predict the antidepressant onset time, and only 10–50% of the animals experience induced effects, which limits the reliability and practicality of the model [10]. CUMS is currently the most commonly used animal model of depression. It can mimic the onset environment of depression in real life, and studies have found that CUMS is related to lipid metabolism and glutamate metabolism, but this model requires a long study time and heavy workload, and so it is not suitable for screening a large number of antidepressant drugs [11,12]. Drug models, such as reserpine models, can be used as simple and economical animal models for antidepressants. Importantly, a previous study found that chronic treatment with reserpine reduced 5-HT levels in the brains of rodents, supporting the use of reserpine as an animal model of progressive depression in rodents [13]. However, the structure of the cerebral cortex of rodents is quite different from that of humans, and high-throughput screening cannot be achieved. Therefore, it is necessary to explore model organisms and models suitable for high-throughput screening of drugs.

The zebrafish (Danio rerio), which has emerged in recent years, has become a model species for translational research in neuroscience fields, including depression [14,15,16]. Due to their physiology (neuroanatomy, neuroendocrine, neurochemistry) and the genetic homology of mammals, robust phenotypes, low cost, fast reproduction cycle, high-throughput genetic value, zebrafish has become the experimental model for studying depression [17]. In addition, zebrafish are highly sensitive to commonly used psychotropic drugs. Zebrafish provide an important perspective for drug screening for depression. Recent studies have successfully applied CUMS to zebrafish to explore depression-like states and changes to the brain proteome profile and neurogenesis, the results showed memory deficits and elevated cortisol levels caused by CUMS, which are similar to depression-like states in humans and rodents [18,19,20]. It has been reported that after the stress with reserpine, the locomotor ability and exploratory behavior of zebrafish were reduced, the levels of cortisol were increased, and 5-HT and norepinephrine were decreased [21], which also simulated the state of human depression.

Compared with traditional research methods, metabolomics detects small molecular metabolites in samples, reflects the overall level of these small molecular metabolites, and identifies specific biomarkers, thus revealing related metabolic pathways [22,23]. Emotion and cognitive function are the manifestation of brain function, and the level of its metabolites can reflect the body’s central nervous function to a certain extent. Metabolomics has been widely used in the study of brain diseases, which can directly reflect the trend of pathological changes in the brain and the effect of drug intervention through the dynamic change rule of metabolite content in the brain tissue and biological fluid.

In this article, we established the CUMS model and reserpine model in mice and zebrafish, respectively. The effects of CUMS and reserpine were preliminarily analyzed through behavioral tests and brain pathology sections, and then compared metabolic differences through metabolomics. The results showed that the reserpine-induced zebrafish depression model is a reliable model for high-throughput screening of antidepressants.

## 2. Results

### 2.1. Effect of CUMS on Behavior and Brain Morphology of Mice and Zebrafish

**Figure 1:**
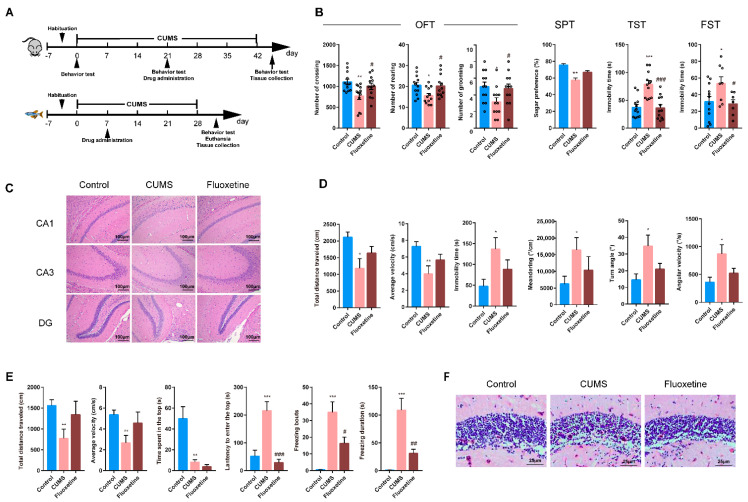
Experimental design and effect of CUMS on mice and zebrafish. (**A**) Experimental design for CUMS. (**B**) The results of behavioral tests for OPT, SPT, FST and TST of CUMS mice. The round symbols indicate data distribution. (**C**) Histopathological observation on mice hippocampus, 100×. (**D**,**E**) The results of behavioral tests for OFT and NTT of CUMS zebrafish. (**F**) Histopathological observations on adult zebrafish brain, 200×. Data are represented as mean ± SEM. * *p* < 0.05, ** *p* < 0.01, *** *p* < 0.001, compared with control; # *p* < 0.05, ## *p* < 0.01, ### *p* < 0.001, compared with CUMS.

The effect of CUMS on mice and zebrafish are shown in Figure 1. The CUMS mice showed depression-like behavior (Figure 1B), which was manifested as the reduction in the number of the crossing, rearing and grooming in the open field test (OFT), the increase in the immobility time of tail suspension test (TST) and forced swimming test (FST), and the decrease in sugar preference. Additionally, CUMS significantly reduced the cells in the hippocampus of mice, with irregular cell morphology, enlarged gaps, disordered arrangement, and almost disappeared cell nuclei (Figure 1C). After three weeks of fluoxetine treatment, the depression-like behavior of the mice improved and the morphology of brain cells returned to normal.

Then, we established a CUMS model on the zebrafish. The results showed that CUMS significantly reduced the locomotor ability, including significantly reduced the total distance and increasing the immobility time (Figure 1D). In the novel tank test (NTT), CUMS significantly reduced the exploratory behavior of zebrafish, including decreased time spent in the top, increased latency and freezing duration (Figure 1E). Similarly, the periglomerular gray zone (PGz) of CUMS zebrafish also showed cells reduced with irregular cell morphology, enlarged gaps and disordered arrangement (Figure 1F). After three weeks of fluoxetine treatment, the locomotor ability and exploratory behavior were improved, and the PGz cells were arranged tightly and regularly. These results indicate that under CUMS conditions, zebrafish can replace mice in screening for antidepressants, highlighting that zebrafish is a suitable model organism.

### 2.2. Effect of Reserpine on Behavior and Brain Morphology of Mice and Zebrafish

**Figure 2:**
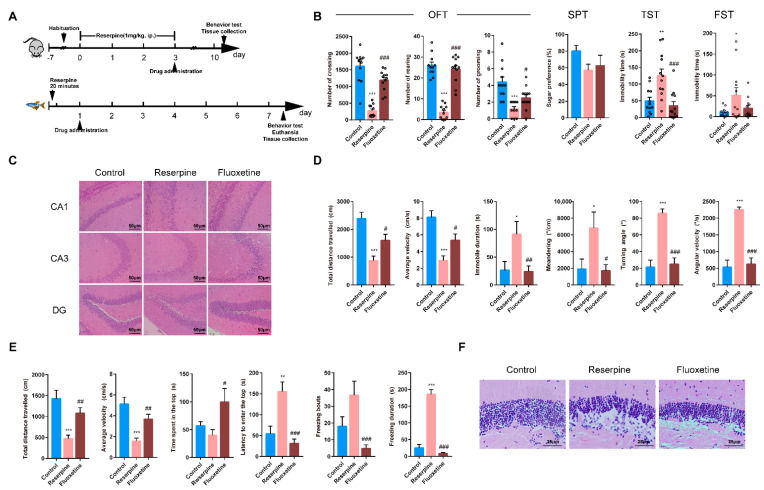
Experimental design and effect of reserpine on mice and zebrafish. (**A**) Experimental design for reserpine. (**B**) The results of behavioral tests for OPT, SPT, FST and TST of reserpine in mice. The round symbols indicate data distribution. (**C**) Histopathological observation of mice hippocampus, 200×. (**D**,**E**) The results of behavioral tests for OFT and NTT of reserpine in zebrafish. (**F**) Histopathological observations on adult zebrafish brain, 200×. Data are represented as mean ± SEM. * *p* < 0.05, ** *p* < 0.01, *** *p* < 0.001, compared with control; # *p* < 0.05, ## *p* < 0.01, ### *p* < 0.001, compared with CUMS reserpine group.

The schedule for the experimental procedure is provided in Figure 2A. The results obtained are similar to the results of the CUMS model. After intraperitoneal injection of reserpine, mice showed depression-like behavior such as decreased locomotion ability, significantly increased immobile time of FST and TST (Figure 2B) and abnormal hippocampal morphology (Figure 2C). After one week of fluoxetine treatment, the locomotor ability of the mice was restored to a certain extent, and the cells in the CA1, CA3 and DG regions of the hippocampus were stained darker and arranged regularly and densely.

According to previous reports, we selected the concentration of reserpine as 10 mg/L to stimulate zebrafish. On this basis, we explored the administration time of the positive drug fluoxetine. We first tracked the behavior of zebrafish for seven consecutive days (Supplementary Figure S1A,B), and observed the brain morphology (Supplementary Figure S1C), we found that 24 h after reserpine was given, a depressive phenotype appeared, and on the seventh day, the zebrafish was completely depressed (Supplementary Figure S2). We also found that reserpine can quickly induce depression-like behavior in zebrafish, including significantly reduced the locomotor ability and the locomotor ability (Figure 2D,E) and cause morphological changes in the brain (Figure 2F). When fluoxetine was given after complete depression, it was found that fluoxetine did not improve depression-like behavior in zebrafish, and that when fluoxetine was given in the appearance of depressive phenotype, it can improve the depressed phenotype. These results showed that reserpine-induced zebrafish depression model is consistent with the CUMS zebrafish model in behavioral and pathological aspects.

### 2.3. Metabolic Analysis

**Figure 3:**
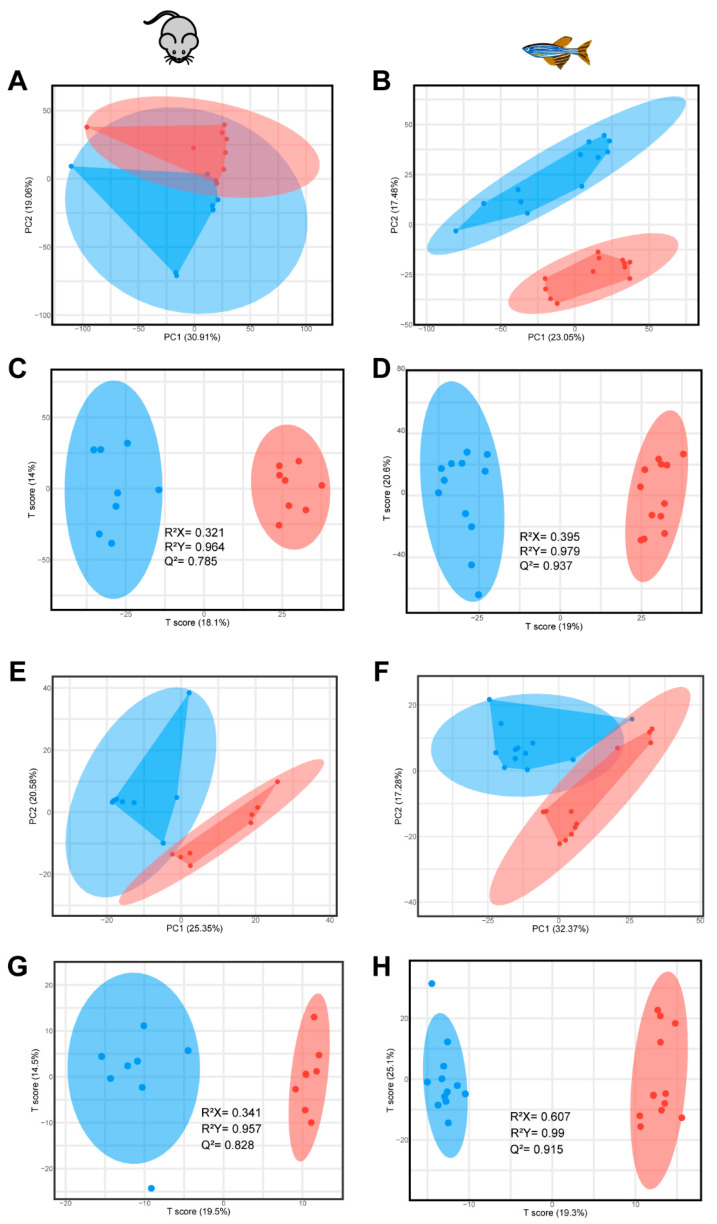
The PCA and OPLS-DA score plots of each model. (**A**) The PCA score plot of CUMS in mice. (**B**) The PCA score plot of CUMS in zebrafish. (**C**) The OPLS-DA score plot of CUMS in mice. (**D**) The OPLS-DA score plot of CUMS in zebrafish. (**E**) The PCA score plot of reserpine in mice. (**F**) The PCA score plot of reserpine in zebrafish. (**G**) The OPLS-DA score plot of reserpine in mice. (**H**) The OPLS-DA score plot of reserpine in zebrafish. Blue represents Control group, red represents model group.

Metabolic profiling was conducted according to the chromatographic and mass spectrum conditions described above. A total of 3409 (2498 in positive mode and 911 in negative mode), 1891 (614 in positive mode and 1277 in negative mode), 2210 (1259 in positive mode and 951 in negative mode), 2543 (1621 in positive mode and 922 in negative mode) features were obtained in the brain in CUMS-induced mice model, CUMS-induced zebrafish model, reserpine-induced mice model and reserpine-induced zebrafish model, respectively. According to the PCA score plots displayed in Figure 3, a clear separation between two groups was observed in each model. To further analyze the differences in metabolic profiles between groups, an OPLS-DA score plot was produced for individual depression models, both R2Y and Q2 were greater than 0.5, suggesting that the models were predictive.

**Figure 4:**
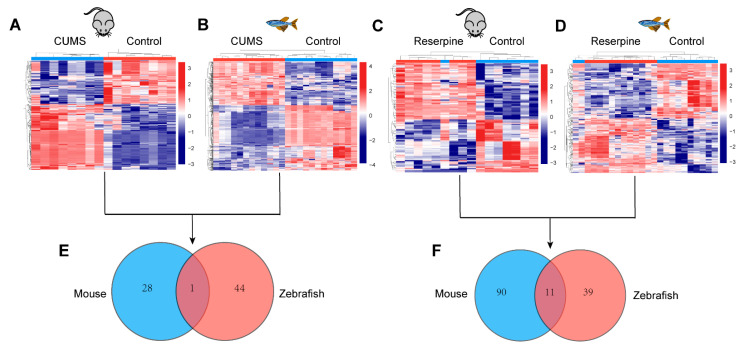
The heatmap and Venn diagram of each model. (**A**) The heatmap of CUMS in mice. (**B**) The heatmap of CUMS in zebrafish. (**C**) The heatmap of reserpine in mice. (**D**) The heatmap of reserpine in zebrafish. (**E**) Venn diagram of differential metabolites that were in common with or unique to the CUMS models in mice and zebrafish. (**F**) Venn diagram of differential metabolites that were in common with or unique to the reserpine models in mice and zebrafish. In (**A**–**D**), Blue represents Control group, red represents model group.

**Figure 5:**
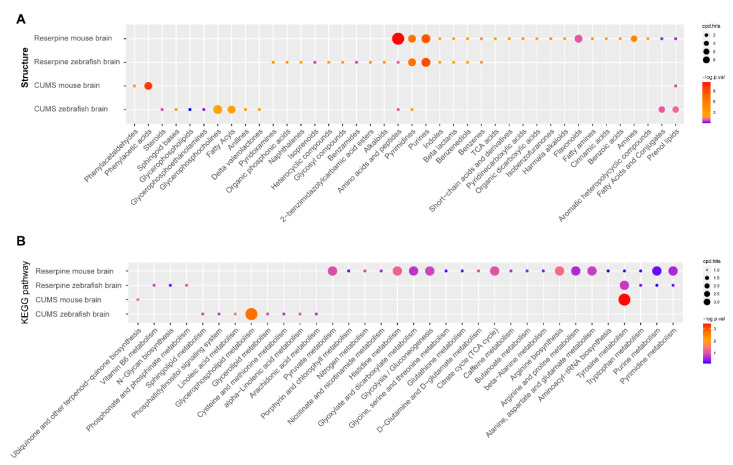
Metabolic analysis results of the four depression models. (**A**), the structure types of the metabolites. (**B**), the pathways of the metabolites.

Clustering of metabolic disturbances in each depression model are shown in Figure 4A–D. Based on the criteria of VIP > 1 and FDR < 0.05, a total of 28,44,90 and 39 different metabolites were identified in these models (Tables S1–S4). Additionally, the metabolic overlap between the two conditions was explored, as shown in Figure 4E; in the CUMS models, there is only one different metabolite overlap between mice and zebrafish, Melibiitol, which can be used as a nutrient component and membrane stabilizer to participate in lipid metabolism and galactose metabolism pathways in vivo. Additionally, as shown in Figure 4F, in the reserpine models, there are 11 overlapping metabolites, including Ginkgolide A, indoleacetic acid, 4-hydroxybenzaldehyde, 5-methoxytryptamine, adenine, tyramine, 2-methoxybenzoic acid, methomyl, 4’-methoxychalcone, leucoharmine and norepinephrine. In addition, the structure types and pathways of unique metabolites are shown in Figure 5, the depression models induced by CUMS have fewer metabolic types than the reserpine-induced depression models. Additionally, as shown in Figure 4B, the metabolic pathways of reserpine-induced mice depression model mainly focused on amino acid metabolism, purine metabolism, pyrimidine metabolism, and tricarboxylic acid cycle; the metabolic pathways of the reserpine-induced zebrafish depression model and CUMS mice model mainly focused on tyrosine metabolism; the CUMS zebrafish model metabolic pathways focused on glycerophospholipid metabolism.

## 3. Discussion

Depression is a mood disorder characterized by feelings of unpleasantness, helplessness, sadness, and despair. Mild cases manifest as poor mood, lack of interest, self-blame, decreased self-evaluation, and fatigue, often accompanied by symptoms such as decreased appetite and libido, early awakening and weight loss, and severe cases often have suicidal tendencies [3]. As serotonin reuptake inhibitors (SSRIs) have a different structure and pharmacological mechanism than previously used tricyclic antidepressants, with clear curative effects, fewer adverse reactions, and safe application, they have rapidly become the first-line clinical antidepressants in recent years. It is currently the most important class of antidepressants. Fluoxetine is the first discovered selective serotonin reuptake inhibitor (SSRI) and the most widely used antidepressant in the world. Fluoxetine plays an antidepressant role mainly by blocking the reuptake of 5-HT and enhancing the release and transmission of 5-HT, with little effect on norepinephrine and dopamine [24]. Studies have found that fluoxetine can increase serotonin levels in brain regions such as the prefrontal cortex, hippocampus and synaptic spaces in the striatum, and improve patients’ moods. Therefore, fluoxetine was selected as the positive drug in this paper.

According to reports, in rodents, reserpine can cause hypofunction, motor stereotypes, dyskinesias, lethargy, and anhedonia [25,26,27,28,29]. After intraperitoneal injection of reserpine for three consecutive days, the mice showed symptoms of reduced mobility, desperation and anhedonia, which is consistent with the results reported in the literature, indicating that the model was successfully constructed. In addition, H&E staining of hippocampal tissue revealed that reserpine reduced the number of hippocampal CA1, CA3, and DG cells and arranged them loosely. After fluoxetine treatment, the number of hippocampal CA1, CA3, and DG cells increased and arranged regularly. These results were consistent with CUMS model results, suggesting that reserpine-induced depression-like phenotypes are similar to those induced by CUMS. In order to improve screening efficiency and conduct high-throughput screening, we established reserpine-induced models on zebrafish. It has been reported in the literature that 20 and 40 mg/L reserpine did not cause significant acute behavioral effects, and locomotor ability was significantly reduced after 7 days, similar to the locomotor retardation observed in depression [30,31,32,33]. Based on this research, we found that when the concentration of reserpine was 10 mg/L, it could cause zebrafish to show reduced locomotor ability and exploratory behavior. Analysis of the pathological characterization of zebrafish brain revealed that reserpine caused a certain degree of damage to PGz, which was similar to the pathological results of the CUMS model zebrafish. The above results suggest that the zebrafish depression model induced by reserpine has similar behavioral and pathological features to the CUMS zebrafish model. Subsequently, we conducted metabolomics analysis on the brain tissues of mouse and zebrafish under the two stress modes and found that there was a significant separation between the control group and the model group. Additionally, a number of differential metabolites were identified. In the CUMS models, only one metabolite, Melibiitol, was shared by mouse and zebrafish, while in the reserpine models, there were 11 metabolites shared by mouse and zebrafish, indicating the similarity between zebrafish and mouse in the reserpine model. In addition, from a time perspective, CUMS took a long time, requiring four to seven weeks, the reserpine mouse model required 10 days, and the reserpine zebrafish model only took 7 days. The above results preliminarily suggest that reserpine-induced zebrafish depression model is feasible.

From the above results, it can be seen that zebrafish can show similar behaviors to rodents and take a short time, indicating that zebrafish is a reliable model organism for studying depression. The reserpine-induced zebrafish depression model can not only achieve rapid and high-throughput screening of drugs, but is also closer to the mice model, indicating that the reserpine-induced zebrafish depression model is reliable and effective. However, from the perspective of the pathological mechanism, the depression model induced by reserpine is established on the basis of the monoamine hypothesis, and the mechanism of depression is complicated and cannot be fully explained by the monoamine hypothesis alone. With the development of the study for the pathogenesis of depression, new targets and new mechanisms will be identified, and the reserpine-induced zebrafish depression model is not suited for screening the new-target drugs.

## 4. Materials and Methods

### 4.1. Animals

C57BL/6 mice, male, weighing 18–22 g, were commercially purchased from Qinglongshan Animal Farm, and kept at a laboratory animal barrier system with required environment (temperature of 24 ± 1 °C, relative humidity of 45 ± 15%, and a 12 h light/dark cycle). Food and water were readily available throughout the experiment, except where specified. The experiments began after 1 week of habituation to the housing conditions.

Zebrafish (AB strain) were maintained in a fish-farming system at the China Pharmaceutical University-Shandong Ruiying Group Joint Laboratory. The zebrafish feeding method was carried out according to The Zebrafish Book [34]. The room temperature was maintained at 28.5 °C on a constant light cycle (14 h light/10 h dark), and the water (KCl 0.05 g/L, NaHCO_3_ 0.025 g/L, NaCl 3.5 g/L, and CaCl_2_ 0.1 g/L, purchased from Sinopharm Chemical Reagent Co., Ltd., Shanghai, China) was circulated continuously. The zebrafish were fed freshly hatched brine shrimp twice daily. Less than 5 adult zebrafish were in the breeding tank. All experiments were approved by ethics Committee of China Pharmaceutical University.

### 4.2. CUMS Model

The CUMS paradigm on mice was adapted from past research. In total, 11 different stressors were presented randomly twice a day for a total of 49 consecutive days [35,36,37]. Stressors including cold water swimming (5 min), room temperature swimming (5 min), and tail suspension (15 min), food deprivation (24 h), lack of water (24 h), cage tilt (30 degrees, 24 h), wet litter (250 mL of water on the litter bed, 24 h), cage shaking (15 min), inversion during the day and night (24 h), being restrained (2 h), clipped tail (2 cm from the tip of the tail, 2 min).

The CUMS paradigm on zebrafish was followed previous studies. Stressors were presented randomly twice a day for a total of 28 days to avoid habituation [38,39,40]. The stressors included low water to expose the dorsal body wall to the air (2 min), crowding in a 250 mL beaker containing only 150 mL of water (30 min), chasing with a net (8 min), cooling the water to 23 °C, heating the water to 33 °C (30 min), tank change, restraint stress and predator stress. All stressors were applied between 08:30 a.m. and 17:00 p.m. The schedule for the experimental procedure is provided in Figure 1A.

### 4.3. Reserpine Model

After behavioral screening, 36 mice were randomly divided into 3 groups, namely the control group, reserpine group and fluoxetine group. The model group and fluoxetine group were injected intraperitoneally with reserpine (Sigma-Aldrich (Shanghai) Trading Co., Ltd., Shanghai, China) at a dose of 1 mg/kg for 3 days. Reserpine was dissolved in glacial acetic acid (Xilong Science Co., Ltd., Shenzhen, China) and diluted with distilled water to a final concentration of 0.5% acetic acid. The volume is 0.2 mL. The fluoxetine (Shanghai Huyuan Pharmaceutical Co., Ltd., Shanghai, China) group was administered at a dose of 20 mg/kg/day.

Reserpine-induced zebrafish depression model was to expose zebrafish to 10 mg/L reserpine solution for 20 min. According to our previous results, fluoxetine was given 24 h later, and the concentration was 0.1 mg/L. The schedule for the experimental procedure is provided in Figure 2A.

### 4.4. Behavior Test

The open field test (OFT), sucrose preference test (SPT), forced swimming test (FST), and tail suspension test (TST) were conducted as previously described [41,42,43]. Spatial exploration behavior in mice was tested by the OFT, and the crossing, rearing and grooming were measured during the 5 min session. The SPT means the ratio of consumed 1% sucrose solution relative to that of total solution in the test and was used as a measure of anhedonia in mice. The duration of the FST and TST was 6 min, and the immobility time was recorded during the last 4 min. The OFT and novel tank test (NTT) were used to assess depression response in experimental zebrafish [44,45]. Noldus software and SONY SSC-DC578P camera were used for the automatic tracking of zebrafish behavior.

### 4.5. Hematoxylin-Eosin Staining (HE)

The embedded tissue wax blocks were sliced with a thickness of 4 μm, adhered to the slides, and dried at 45 °C. Tissue sections were dewaxed with xylene. After soaking and staining with hematoxylin for 5 min, rinse with distilled water. Then, 75% hydrochloric acid ethanol differentiated for 30 s; rinse with distilled water for 10 min; eosin staining for 2 min; dehydration; neutral resin sealing; microscope observation and photography [46]; reagents were purchased from Shenggong Biological Engineering (Shanghai) Co., Ltd., Shanghai, China.

### 4.6. Sample Collection and Preparation

After the behavioral test, the animals were sacrificed and the brains were dissected on ice, weighed, and frozen. Prior to analysis, all samples were thawed at room temperature. In total, 100 mg of brain tissue was transferred to a 2 mL centrifuge tube and mixed with pre-cooled acetonitrile, methanol ( Merck Chemical Technology (Shanghai) Co., Ltd., Shanghai, China) and water (V:V:V = 9:4:2). The mixture was homogenized until the tissue was broken and allowed to stand for an hour, then centrifuged at 13,300 rpm for 20 min at 4 °C. The supernatant was evaporated until dry, and the residue was reconstituted with 100μL of ACN/H2O (V:V = 50:50) and then centrifuged at 13,300 rpm for 20 min at 4 °C, and the supernatant was taken in a sample vial for further analysis. In total, 80 μL of the supernatant was collected, and the remainder was mixed as QC samples.

### 4.7. LC-MS Analysis

Chromatographic separation was performed on a high-performance liquid chromatography-quadrupole time-of-flight mass spectrometer (Agilent1260, Agilent, Santa Clara, CA, USA) equipped with a Waters XSelect HSS T3 column (2.1 × 100 mm, 2.5 μm), and the column temperature was maintained at 40 °C. The mobile phase consists of 0.1% formic acid (A) (Fisher, Waltham, MA, USA) and acetonitrile (B) and the gradient operated at a flow rate of 0.4 mL/min by eluting in 98% A for two minutes, then increasing solvent B to 100% within 18 min, and eluting in 100% solvent B for five minutes. The autosampler temperature was set at 4 °C and the injection volume per sample was 2 µL. MS data were obtained by a mass spectrometer equipped with an electrospray source (ESI) in both positive and negative modes, and the sweep range was: 50–1000 *m*/*z*. For positive and negative modes, the operating parameters were set as follows: gas temperature of 325 °C, gas flow of 8 L/min, nebulizer pressure of 40, sheath gas temp of 350 °C, sheath gas flow of 12 L/min, nozzle voltage of 0 V when positive and 500 V when negative, Fragmentor of 130 V, Skimmer of 65 and Octopole RF Peak of 750.

### 4.8. Data Analysis

All data from behavioral test are expressed in the form of mean ± standard deviation. The statistical analysis was carried out by using Graphpad Prism7.0. The results were analyzed by one-way analysis of variance (ANOVA). The significance level was set at *p* < 0.05.

LC-MS data files including MS1 and MS2 spectra data were converted to mzXML format files using the MS Convert program in the Proteowizard software (Version: 3.0.19291-5e92459cc) for further analysis. Using xcms R package (Version: 3.10.2) for retention time correction and ion peak matrix extraction. The MetaboAnalystR R package (Version: 3.0.3) was used for quality control. After pretreatment, a table consisting of sample name, peak area, *m*/*z*, retention time, etc. was obtained. Then, the data were imported to RStudio for analysis. The variables with VIP values larger than 1.0 and *q*-values less than 0.05 were deemed to be statistically significant.

## Data Availability

Not applicable.

## Supplementary Materials

The following are available online at https://www.mdpi.com/article/10.3390/ijms22179505/s1.
