# Supplementary material for: A Reliable High-Throughput Screening Model for Antidepressant
Source: Int J Mol Sci. 2021 Sep 1;22(17):9505. doi: 10.3390/ijms22179505 (PMC8430800; doi:10.3390/ijms22179505)
Supplement: Supplementary file 1 [file ijms-22-09505-s001.zip › ijms-1330174-supplementary.pdf]

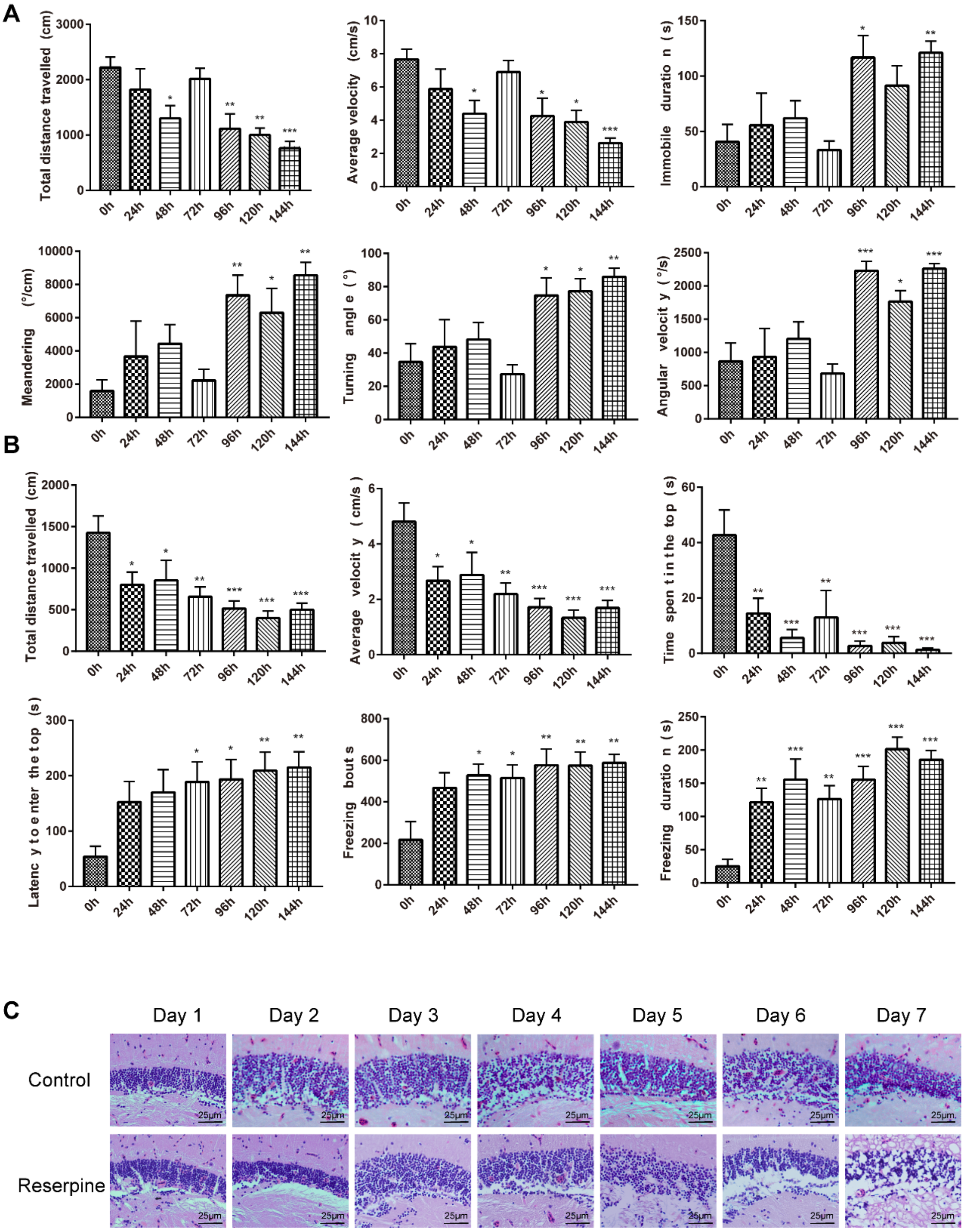

Supplement Figure S1. Changes in behavior and brain cell morphology of zebrafish for 7 consecutive days. A, OFT results of zebrafish for 7 consecutive days. B, NTT results of zebrafish for 7 consecutive days. C, HE staining results of the control group and reserpine group for consecutive 7 days, 200×. Data are represented as mean ± SEM. * $p < 0.05$, ** $p < 0.01$, *** $p < 0.001$, compared with 0h.

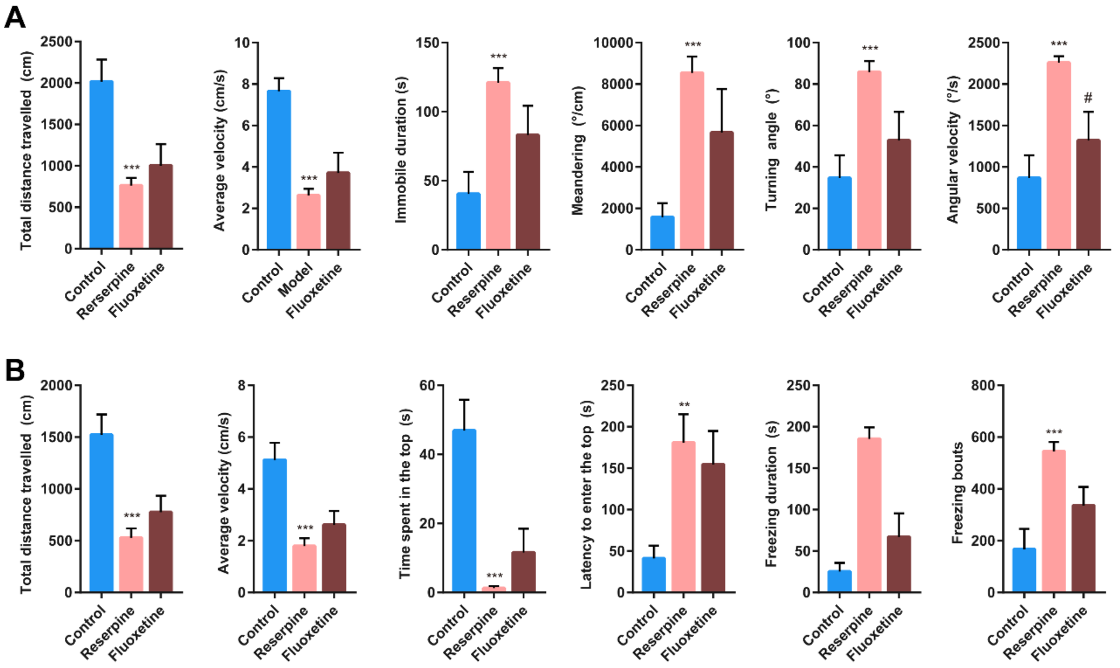

Supplement Figure S2. Exploration of the time point of administration of positive drugs. A, OFT results of fluoxetine administration after complete depression. B, NTT results of fluoxetine administration after complete depression. Data are represented as mean ± SEM. * $p < 0.05$, ** $p < 0.01$, *** $p < 0.001$, compared with control group.

Table S1. Potential biomarkers in the brain profile and their changes of CUMS mice model

**Metabolite**	***m/z***	**VIP Score**	**Model vs. Control**	
			**Fold Change**	**q Value**
L-Glutamic acid	146.0453	2.248731	1.2794	0.002134
Glutathione-SH	308.0924	1.635312	2.635	0.002379
Succinic acid	117.0190	2.588976	1.9652	3.01E-05
Flavin Mononucleotide	456.1032	2.095344	6.1546	4.86E-05
Inosine	271.0844	1.943672	3.7929	0.000141
2-Hydroxyestrone	219.1770	1.865479	2.7527	0.000369
5-Aminoimidazole ribonucleotide	298.059`	1.794017	1.5852	0.00041
N-Glycolylneuraminic acid	308.0964	1.922736	2.4872	0.000644
Paraxanthine	183.0684	1.960662	1.4144	0.000656
Pyridoxamine	101.1096	1.628334	2.0827	0.001131
CMP-N-trimethyl-2-aminoethylphosphonate	457.0991	1.876887	8.0945	0.001245
Melibiitol	429.1022	1.679492	1.5315	0.001296
3-Sulfinoalanine	152.0016	2.2276	1.6965	0.001318
Oxoglutaric acid	145.0135	2.309281	1.8418	0.001364
Phenylpyruvic acid	209.0446	2.22746	1.2062	0.001691
1-Methylnicotinamide	102.0571	1.796312	1.315	0.00173
Sphingosine 1-phosphate	308.2355	1.676835	3.5687	0.001772
3-Methyl-1-hydroxybutyl-ThPP	484.1291	1.660777	2.8564	0.00184
N-Acetylaspartylglutamic acid	307.0924	1.672564	2.611	0.001878
Tetrahydrofolyl-[Glu](2)	613.1758	1.649844	3.6391	0.002014
O-Phosphoethanolamine	141.0189	1.778278	2.8027	0.00249
Agmatine	215.0908	1.795952	1.242	0.002595
6-Hydroxymelatonin	84.04675	1.570902	1.8169	0.003135
4-Hydroxyphenylpyruvic acid	164.0233	1.595569	2.0865	0.003716

(S)-allantoin	159.0508	1.553718	1.4769	0.004084
cis-Aconitic acid	157.9967	1.709965	1.2958	0.004685
N-Formyl-L-aspartate	55.01926	1.881396	2.4393	0.000181
Formiminoglutamic acid	177.0689	1.691638	1.2803	0.005392

Table S2. Potential biomarkers in the brain profile and their changes of CUMS zebrafish model.

Metabolite	*m/z*	VIP Score	Model vs. Control	
			Fold Change	q Value
Choline	141.0553	1.378513	1.6105	0.004511
L-Histidine	136.0519	1.40153	1.4336	0.002844
Adenine	136.0443	1.533267	2.6774	0.00013
L-Valine	118.0686	1.392658	1.1089	0.005438
Ketoleucine	131.0524	1.663841	1.2628	0.000141
Betaine	118.0686	1.392658	1.1089	0.005438
Spermine	237.1835	1.393166	1.4503	0.001922
Sphinganine	382.2143	1.853979	1.1812	1.89E-05
Deoxycytidine	286.1033	1.305207	1.488	0.00559
L-fucose	165.0577	1.305438	1.2816	0.010151
Acetylcholine	167.0935	1.401139	1.2669	0.004269
Ethanolamine	60.04575	1.412301	1.4238	0.001647
Methylimidazole acetaldehyde	124.0594	1.304143	1.2006	0.003328
2-Hydroxyglutarate	149.0262	1.4036	1.1981	0.000917
1-Methylhistamine	162.0605	1.082552	1.4144	0.012955
Epinephrine	184.0793	1.49599	1.1509	0.001339
N-Acetylornithine	189.0887	1.520697	1.4882	0.000627
3,4-Dihydroxymandelic acid	221.0034	1.395848	1.0857	0.001909
N(omega)-Hydroxyarginine	269.0247	1.609238	1.6138	0.000682
Arachidic acid	312.2989	1.94432	1.6426	1.61E-07
(GlcN)1 (Ino(acyl)-P)1 (Man)1	322.2821	1.678973	1.1892	0.000148
Melibiitol	325.1128	1.204791	1.3168	0.010776
(Gal)1 (Xyl)1 (Ser)1	334.32	1.444281	1.5342	0.001996
Menaquinol	350.213	2.033358	1.795	1.57E-07
Retinal	363.1312	1.4515	4.8478	0.001102
(Gal)1 (GalNAc)1 (Ser/Thr)1	364.2916	2.090529	4.3923	5.79E-09
17alpha,21-Dihydroxypregnenolone	385.1766	1.748318	1.1893	1.74E-05
8,11,14-Eicosatrienoic acid	387.173	1.820401	1.1346	7.78E-06
7-a,25-Dihydroxycholesterol	418.3412	1.90794	3.3141	2.26E-06
3alpha,7alpha,12alpha-trihydroxy-5beta-cholestan-26-oate	430.3082	1.896109	3.1546	2.57E-06
5-Dehydroavenasterol	431.3294	1.979759	3.9895	1.36E-07
Avenasterol	433.3449	1.867237	2.0687	2.84E-07
L-Palmitoylcarnitine	436.299	1.98938	2.7529	1.65E-07
4a-Carboxy-4b-methyl-5a-cholesta-8,24-dien-3b-ol	442.3422	2.096404	4.119	3.59E-09
Allocholic acid	453.2871	2.127781	2.1938	2.51E-09
Vitamin K1 2,3-epoxide	466.3399	1.948566	4.0957	1.85E-06
2-MeOE1 3G	476.1998	1.315972	2.0515	0.006698
Retinoyl b-glucuronide	477.2281	1.546462	1.6094	0.000172
Taurochenodesoxycholic acid	500.2882	1.672458	1.439	8.31E-05
Phylloquinol	511.3763	1.867364	1.6499	1.98E-06
(GalNAc)1 (GlcNAc)1 (Man)1 (P)1 (Ser/Thr)1	685.45	1.554032	2.204	0.000306

(GalNAc)1 (GlcNAc)1 (Man)1 (Rib-ol)1 (P)2 (Ser/Thr)1	899.5447	1.669647	2.0022	8.50E-05
all-trans-Decaprenyl diphosphate	903.5697	1.375899	2.3072	0.002934
Calcitriol	416.3265	1.945708	3.1914	1.06E-06

Table S3. Potential biomarkers in the brain profile and their changes of reserpine mice model.

Metabolite	*m/z*	VIP Score	Model vs. Control	
			Fold Change	q Value
Oxalacetic acid	131.0699	1.13953	2.307	0.001068
Ornithine	131.0346	1.496262	0.14063	0.048511
Ginkgolide A	135.0316	1.011941	0.399969	0.002109
4-Hydroxybenzoic acid	137.0237	1.291823	0.12915	1.76E-07
L-Glutamic acid	146.0459	1.719314	0.491659	0.00357
4-Methylbenzoic acid	155.0072	1.43633	0.17228	0.002315
Hypoxanthine	155.0466	1.667787	0.032973	9.25E-05
L-Aspartic acid	170.0463	1.034148	0.278299	0.019391
Indoleacetic acid	174.0844	1.607957	0.060147	7.10E-05
4-Methoxycinnamic acid	176.9347	1.114404	0.377909	0.007763
4-Hydroxybenzaldehyde	181.0505	1.245837	0.32557	0.004281
Xanthurenic acid	186.0456	1.540846	0.304761	0.000553
Homo-L-arginine	187.1085	1.43564	0.202749	0.002258
Indole-3-methyl acetate	188.0924	1.241937	0.31247	0.003961
4-Guanidinobutanoic acid	190.0719	1.489341	0.083079	0.00132
5-Hydroxyindoleacetic acid	190.051	1.57496	0.303269	0.000342
Cysteine-S-sulfate	200.0567	1.326174	0.10726	0.003515
Melatonin	213.0865	1.39476	0.427021	0.000798
N-Acetylleucine	218.0672	1.387254	0.323891	0.001388
Suberic acid	219.0709	1.565075	0.067437	0.00065
Zeatin	256.0567	1.31965	0.384861	0.002644
Pinocembrin	301.0363	1.31419	0.324311	0.005532
Glutathione	352.125	1.462996	0.18017	0.001716
Hexylamine	106.1114	1.623549	4.2536	3.85E-05
Histamine	112.0502	1.265141	4.0511	0.00287
Theobromine	113.0341	1.258999	2.3654	0.003638
5-Methoxytryptamine	118.0863	1.503989	4.6666	0.000618
Isokobusone	123.0563	1.157759	3.6837	0.005034
Corticosterone	131.0531	1.551764	2.6915	0.000174
5-Methoxydimethyltryptamine	132.0775	1.448071	2.0971	0.000553
Adenine	136.0745	1.331275	6.9954	0.002057
Tyramine	138.0482	1.248276	3.5862	0.003533
Norspermidine	138.0481	1.437596	4.2337	0.000692
Betaine	141.0154	1.769625	25.705	4.76E-07
Chalconaringenin	148.0048	1.578186	3.0978	9.34E-05
5-Methylcytosine	148.0603	1.26336	2.3284	0.003401
Taurine	148.0604	1.298481	4.1508	0.002699
L-Glutamine	151.0748	1.470277	7.5741	0.000441
Acetaminophen	152.0325	1.169405	3.3975	0.008319
2-Methoxybenzoic acid	153.0409	1.287064	4.3692	0.00268
N-Acetylhistamine	154.0486	1.427078	13.889	0.000833
5-Aminopentanoic acid	159.0279	1.265786	5.7223	0.005117
Indole	159.0281	1.368072	4.713	0.00132
N,N-Dimethylaniline	160.0757	1.242714	3.4172	0.003648

1-Methylnicotinamide	160.0301	1.129919	4.9108	0.009779
6-Methylcoumarin	161.1288	1.411082	2.5849	0.001288
L-Carnitine	162.0588	1.554823	48.538	0.000219
Methomyl	163.1155	1.26588	2.9727	0.003181
2-Amino-3-phosphonopropionic acid	163.115	1.323103	2.4925	0.001496
Phosphoenolpyruvic acid	170.0806	1.388322	90.8	0.005897
1,2,3,4-Tetrahydro-beta-carboline	172.9822	1.43458	3.3033	0.001635
beta-Carboline	175.1112	1.120528	2.8852	0.007044
Citrulline	176.1066	1.2618	43.732	0.011024
Umbelliferone	185.0421	1.354757	12.337	0.001851
Norepinephrine	187.1079	1.466647	0.1345	0.000494
Glycylleucine	189.0058	1.2	3.5473	0.006389
Harmalan	189.1595	1.439714	2.5406	0.000475
Azelaic acid	193.0355	1.464806	7.721	0.000808
Triethanolamine	194.0328	1.494315	7.0542	0.000596
2-(Methylamino)benzoic acid	215.0164	1.791018	132.02	1.67E-08
Cotinine	218.186	1.361567	4.2902	0.001541
N6-Acetyl-L-lysine	227.0832	1.340296	2.1527	0.001851
2(3H)-Benzothiazolethione	230.9904	1.803034	255.17	1.67E-08
Deoxyuridine	233.1145	1.079472	2.3514	0.009378
(2-Naphthalenyloxy)acetic acid	241.1552	1.531716	2.183	0.000415
Homocarnosine	241.0823	1.346207	18.011	0.00083
(E)-Monocrotophos	241.1553	1.523085	2.6841	0.000183
Phosphocreatine	244.0934	1.293719	3.5919	0.002196
Carnosine	249.0542	1.109898	2.5313	0.008733
Phenobarbital	255.0987	1.628626	26.202	7.04E-05
Glucose 6-phosphate	261.1455	1.250669	3.0642	0.00384
4′-Methoxychalcone	261.1449	1.398879	6.1142	0.001124
5-Hydroxy-L-tryptophan	262.1465	1.802219	201.24	4.05E-09
N-Acetylserotonin	263.1688	1.151034	3.7504	0.009983
Uridine	267.0595	1.550593	29.401	0.000286
Deoxycytidine	269.0891	1.2798	4.706	0.002584
Inosine	269.0893	1.114014	5.5799	0.011024
N-Desmethylvenlafaxine	270.092	1.477534	7.0509	0.001111
Norizalpinin	271.0925	1.548818	3.9451	0.000253
Leucoharmine	276.145	1.411904	11.819	0.00079
Norfentanyl	277.1037	1.195822	2.5128	0.004519
8-Hydroxy-deoxyguanosine	284.0996	1.173196	2.6404	0.009195
Adenosine	290.125	1.195205	3.2582	0.00363
N,O-Didesmethylvenlafaxine	291.0715	1.400349	2.6691	0.001347
Avocadyne	307.0816	1.248947	2.2473	0.001942
Dihomo-gamma-linolenic acid	307.5857	1.129729	2.1798	0.004356
Desvenlafaxine	308.0789	1.416499	3.8297	0.000367
Parathion	309.1378	1.342318	4.0732	0.001949
Cinchonidine	312.1316	1.554255	14.47	0.000253
Fexofenadine	524.2743	1.545407	2.4131	0.000121

Table S4. Potential biomarkers in the brain profile and their changes of reserpine zebrafish model.

Metabolite	*m/z*	VIP Score	Model vs. Control	
			Fold Change	q Value
4-Hydroxy-3-(3-methyl-2-butenyl)acetophenone	114.065	1.7508	2.1357	0.001949

Ciliatine	124.0516	1.401687	23.19	0.00189
Phenylacetic acid	134.8933	2.147313	2.8193	1.97E-09
Estrone	136.0477	1.495129	2.0059	0.011024
Adenine	136.0477	1.96408	6.0928	0.000235
Cytosine	144.1374	1.293352	2.653	0.03073
D-Lysine	145.0505	1.6043	0.01723	0.018576
Verapamil metabolite D-617	146.1651	1.351558	2.2026	0.030818
D-Glutamine	147.0454	1.170606	2.2154	0.031831
Phenylethylamine	154.0488	2.303557	4.0908	4.67E-07
Phenyllactic acid	165.0554	1.838931	4.5302	3.67E-05
Phenylglyoxylic acid	169.0134	1.470528	0.030707	0.001562
Tyramine	170.0626	1.957484	2.1036	0.000392
2-Methoxybenzoic acid	170.0808	1.608994	5.627	0.004316
(+)-Calycanthine	174.0385	1.368766	2.1592	0.032685
Indoleacetic acid	174.0875	1.259412	0.7411	0.005456
4-Hydroxybenzaldehyde	181.0702	1.673301	1.3172	0.001428
3-Hydroxyanthranilic acid	186.1123	1.221633	3.4606	0.035528
Ribitol	191.1379	1.247989	3.594	0.024262
Methomyl	201.0149	1.853747	0.61251	0.000804
Norepinephrine	211.1173	1.523521	0.96	0.011589
Juglone	213.1224	1.344809	1.8013	0.018056
Farnesol	229.1003	1.361742	1.9707	0.023684
Undecanoic acid	231.1	1.312904	1.9833	0.019833
5-Methoxytryptamine	235.1648	1.510202	0.47871	0.010743
Leucoharmine	254.1236	2.084792	10.667	0.000102
Fluticasone propionate	262.1645	1.660317	2.0402	0.004852
Carbofuran	263.1381	1.56759	2.0264	0.008477
Girinimbine	264.1439	1.400649	2.755	0.014249
Guanosine	290.0853	1.919581	0.51599	0.000666
4′-Methoxychalcone	302.2184	1.838461	0.23554	0.001227
Deoxyguanosine	312.1547	1.799774	0.13921	0.001326
cis-[8]-Shogaol	327.2304	1.901261	0.20554	0.000511
7-Aminoflunitrazepam	347.2174	1.371047	0.26572	0.022901
Mycophenolic acid	362.0393	1.704051	0.2427	0.000183
Inosinic acid	390.2041	1.721307	2.5634	0.002215
Ginkgolide A	445.1673	2.129507	0.51761	3.98E-05
Deoxycholic acid	451.1705	1.740782	2.4967	0.000109
Glycyrrhetinic acid	477.212	1.752737	0.12234	0.000117
